# New Perspectives on the Electronic and Geometric Structure of Au_70_S_20_(PPh_3_)_12_ Cluster: Superatomic-Network Core Protected by Novel Au_12_(µ_3_-S)_10_ Staple Motifs

**DOI:** 10.3390/nano9081132

**Published:** 2019-08-06

**Authors:** Zhimei Tian, Yangyang Xu, Longjiu Cheng

**Affiliations:** 1 Department of Chemistry, Anhui University, Hefei 230601, China; 2 School of Chemistry and Materials Engineering, Fuyang Normal University, Fuyang 236037, China; 3 School of Social and Public Administration, East China University of Science and Technology, Shanghai 200237, China; 4 Anhui Province Key Laboratory of Chemistry for Inorganic/Organic Hybrid Functionalized Materials, Anhui University, Hefei 230601, China

**Keywords:** Au_70_S_20_(PPh_3_)_12_ cluster, superatom network model, electronic structure, geometric structure

## Abstract

In order to increase the understanding of the recently synthesized Au_70_S_20_(PPh_3_)_12_ cluster, we used the divide and protect concept and superatom network model (SAN) to study the electronic and geometric of the cluster. According to the experimental coordinates of the cluster, the study of Au_70_S_20_(PPh_3_)_12_ cluster was carried out using density functional theory calculations. Based on the superatom complex (SAC) model, the number of the valence electrons of the cluster is 30. It is not the number of valence electrons satisfied for a magic cluster. According to the concept of divide and protect, Au_70_S_20_(PPh_3_)_12_ cluster can be viewed as Au-core protected by various staple motifs. On the basis of SAN model, the Au-core is composed of a union of 2e-superatoms, and 2e-superatoms can be Au_3_, Au_4_, Au_5_, or Au_6_. Au_70_S_20_(PPh_3_)_12_ cluster should contain fifteen 2e-superatoms on the basis of SAN model. On analyzing the chemical bonding features of Au_70_S_20_(PPh_3_)_12_, we showed that the electronic structure of it has a network of fifteen 2e-superatoms, abbreviated as 15 × 2e SAN. On the basis of the divide and protect concept, Au_70_S_20_(PPh_3_)_12_ cluster can be viewed as Au_46_^16+^[Au_12_(µ_3_-S)_10_^8−^]_2_[PPh_3_]_12_. The Au_46_^16+^ core is composed of one Au_22_^12+^ innermost core and ten surrounding 2e-Au_4_ superatoms. The Au_22_^12+^ innermost core can either be viewed as a network of five 2e-Au_6_ superatoms, or be considered as a 10e-superatomic molecule. This new segmentation method can properly explain the structure and stability of Au_70_S_20_(PPh_3_)_12_ cluster. A novel extended staple motif [Au_12_(µ_3_-S)_10_]^8−^ was discovered, which is a half-cage with ten µ_3_-S units and six teeth. The six teeth staple motif enriches the family of staple motifs in ligand-protected Au clusters. Au_70_S_20_(PPh_3_)_12_ cluster derives its stability from SAN model and aurophilic interactions. Inspired by the half-cage motif, we design three core-in-cage clusters with cage staple motifs, Cu_6_@Au_12_(μ_3_-S)_8_, Ag_6_@Au_12_(μ_3_-S)_8_ and Au_6_@Au_12_(μ_3_-S)_8_, which exhibit high thermostability and may be synthesized in future.

## 1. Introduction

Due to the applications in catalysis, optoelectronics, and photoluminescence, ligand-protected gold (Au-L) nanoclusters have drew much attention in both experiment [1,2,3,4,5,6] and theory [7,8,9,10,11]. The synthesis of Au-L clusters contributes much to many areas of science and technology because they have interesting structures [12,13]. In the past few years, the metalloid thiolate-protected Au nanoclusters with µ_3_-S atoms have extended the family and potential applications of Au-L clusters. The experimentally determined metalloid Au-L clusters containing one or two µ_3_-S include Au_21_S(SR)_15_ [14], Au_30_S(SR)_18_ [15,16], Au_38_S_2_(SR)_20_ [17], and Au_103_S_2_(SR)_41_ clusters [18], while Au_30_S_2_(SR)_18_ cluster is a structure from theoretical prediction [19]. A large metalloid Au_108_S_24_(PPh_3_)_16_ cluster with 24 µ_3_-S has been revealed, which consists of an octahedral Au_44_ core, an Au_48_S_24_ shell and 16 Au(PPh_3_) elements [20]. Very recently, Kenzler et al. has synthesized an intermediate size metalloid gold cluster Au_70_S_20_(PPh_3_)_12_, revealing an Au_22_ core surrounded by the Au_48_S_20_(PPh_3_)_12_ shell [21]. According to their report, Au_4_S_4_ unit is a central structural motif in the shell and they suggest that they could not elucidate a definite superatom character or distinct shell structure in the cluster. Thus, it is necessary to give a detailed study for the cluster, which may help to deeply understand the stability and structural nature of the cluster.

Häkkinen et al. proposed the divide-and-protect concept [22], and Au-L clusters are composed of Au-core and staple motifs; Au-core is protected by staple motifs. The concept has been widely used to predict and analyze the structures of Au-L clusters [23,24,25,26,27,28,29,30,31,32]. The idea of staple motif has been introduced since the synthesis of Au_102_(SR)_44_ cluster, and Jadzinsky et al. termed it [1]. To date, various forms of staple motifs (-SR-(AuSR)_x−_) present in experimentally determined and theoretically predicted Au-L clusters. Monomer and dimer staple motifs present in Au_102_(SR)_44_ cluster [1]. Dimer staple motif also presents in Au_36_(SR)_24_ cluster [33]. Bridging -SR ligand and trimer staple motif exist in Au_23_(SR)_16−_ cluster [4]. In addition, gold-thiolate rings present in Au_20_(SR)_16_ and Au_22_(SR)_18_ clusters [8,34]. The protecting motifs include Au and SR, or only SR; moreover, they have two legs. We have predicted a tridentate staple motif with three S legs in the synthesized Au_30_S(SR)_18_ cluster before [19]. According to the superatom complex (SAC) concept proposed by Häkkinen et al [35], the number of valence electrons (V) for Au_m_S_n_(SR)_p_^q^ cluster is computed as bellow: *V* = *m* − 2*n* − *p* − *q*, in which *m*, *n* and *p* are the numbers of Au, S and SR, respectively, whereas *q* is the charge of the cluster. The super shells for spherical Au clusters is |1*S*^2^|1*P*^6^|1*D*^10^|2*S*^2^1*F*^14^|2*P*^6^1*G*^18^|… (*S*–*P*–*D*–*F*–*G*–*H*– denote angular-momentum characters), corresponded to magic numbers 2, 8, 18, 34, 58, …. According to SAC model, clusters with valence electrons 2, 8, 18, 34, 58, … present special stability and they are magic number clusters. The theoretically predicted Au_12_(SR)_9_^+^ and Au_8_(SR)_6_ are 2e magic clusters. Au_25_(SR)_18_^−^, Au_44_(SR)_28_^2−^ and Au_102_(SR)_44_ are 8e, 18e and 58e magic number clusters, respectively. Cheng et al. introduced the superatom-network (SAN) model, which has been used to explore the stability of Au_18_(SR)_14_, Au_20_(SR)_16_, Au_24_(SR)_20_, Au_44_(SR)_28_ and Au_22_(SR)_18_ clusters [8,36,37]. Based on the concept of SAN model, the Au-core of Au-L cluster can be viewed as a network of 2e Au*_n_* (*n* = 3, 4, 5 or 6) superatoms. The interactions between the superatoms are main non-bond interactions.

Here, we investigate the electronic and geometric structure of Au_70_S_20_(PPh_3_)_12_ to obtain deep understanding of it. Based on the superatom complex (SAC) model, this cluster is a 30e compound [35]. The number of valence electrons for Au_70_S_20_(PCH_3_)_12_ cluster does not satisfy the magic number electrons of SAC model. Kenzler et al. reported that the Au core of Au_70_S_20_(PPh_3_)_12_ cluster is Au_22_, and the protecting tetrahedral shell is composed of four Au_4_S_4_ units, four S atoms and 32 gold atoms, and no staple motif presents [21]. We are interested in the synthesized Au_70_S_20_(PPh_3_)_12_ cluster, which has 20 μ_3_-S atoms. Now that the number of the valence electrons does not satisfy the SAC model, why it is stable? How do the 20 μ_3_-S atoms protect the Au-core? What are the protecting motifs of the cluster? With these questions in mind, we tried to analyze the electronic and geometric structure of the cluster using existing theories and models. This work attempts to explain the structure and properties from a new perspective.

## 2. Materials and Methods

We start from the experimental structure of Au_70_S_20_(PPh_3_)_12_ determined as reported by Kenzler et al [21] and the total charge is set to zero. Considering the calculation amount, we used CH_3_ instead of all the Ph ligands, and the structure was then relaxed using the Gaussian 09 software (Revision B 01; *Gaussian*, Inc., Wallingford, CT, USA) [38]. Density-functional theory (DFT) calculations were employed to optimize the geometric structure using Perdew–Burke–Ernzerhof (PBE0) functional [39]. The basis set of Au element is Lanl2dz, while 6-31G * is used for S, P, C, H elements. The molecular orbital (MO) and natural bond orbital (NBO) calculations of Au cores were also carried out at the same level, whereas the basis set of Au element was Lanl2mb. The adaptive natural density partitioning (AdNDP) method was used to analyze the chemical bonding patterns [40]. MOLEKEL software (version 5.4.0.8, Swiss National Supercomputing Centre, Manno, Switzerland) [41] was used to view the chemical bonding patterns. The superatom-network (SAN) model was taken to analyze the chemical bonds in Au_70_S_20_(PPh_3_)_12_ cluster [36].

## 3. Results and Discussion

### 3.1. Geometric Structure

**Figure 1:**
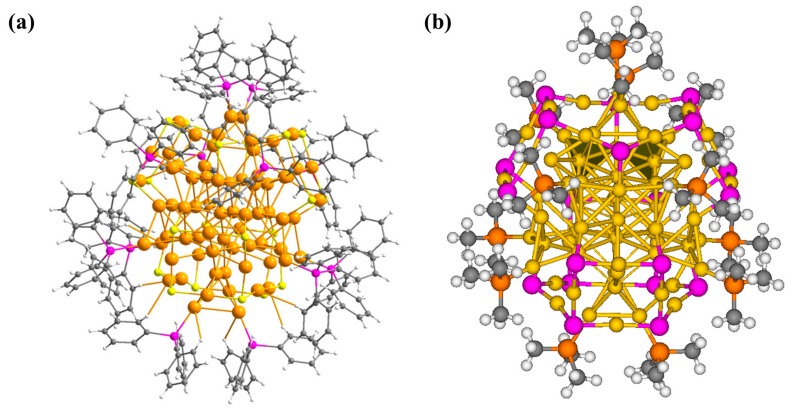
(**a**) Single crystal XRD structure of Au_70_S_20_(PPh_3_)_12_ from [21], reproduced with permission, Royal Society of Chemistry, 2017; (**b**) The optimized structure of Au_70_S_20_(PCH_3_)_12_ cluster. The cluster is obtained at the PBE0/LanL2dz(Au) and 6-31G *(S, C, P, H) level of theory. Au, yellow, S, purple; P, orange; C, gray, H, white.

The structure of the relaxed Au_70_S_20_(PCH_3_)_12_ cluster is given in Figure 1b, which is in D_2_ symmetry. The structural parameters computed here reproduce well with the experimental results.

**Figure 2:**
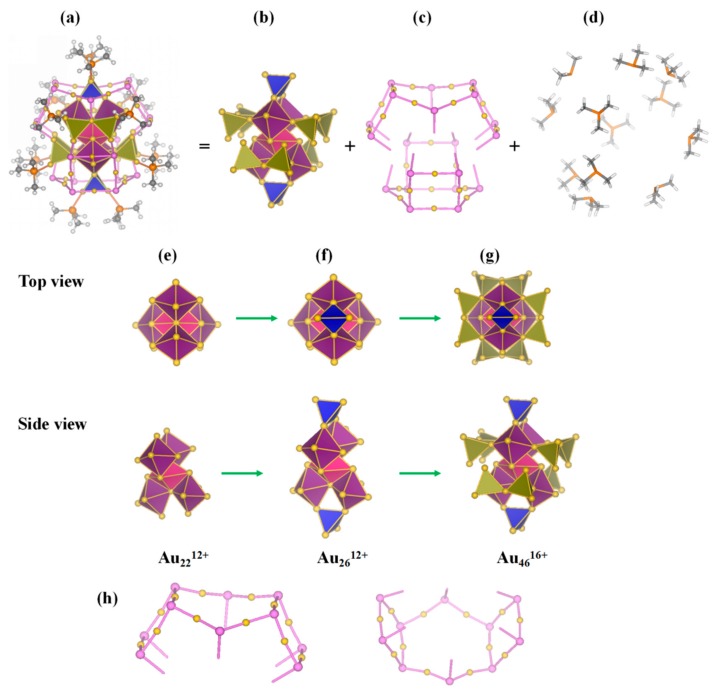
(**a**) Structural model of Au_70_S_20_(PCH_3_)_12_. The [Au_12_(µ_3_-S)_10_]^8−^ and PCH_3_ protecting motifs are given as ball-and-stick models (Au, yellow; P, orange; S, pink; C, gray; H, white). The Au cores are shown as polyhedra. (**b**) Model of Au_46_^16+^ core, (**c**) Two [Au_12_(µ_3_-S)_10_]^8−^ six-tooth staple motifs, (**d**) Model of twelve PCH_3_ protecting motif, (**e**) 6 × 2e SAN of Au_22_^12+^ core, (**f**) Au_26_^12+^ core, (**g**) Au_46_^16+^ core, (**h**) Two views of [Au_12_(µ_3_-S)_10_]^8−^ staple motif.

**Figure 3:**
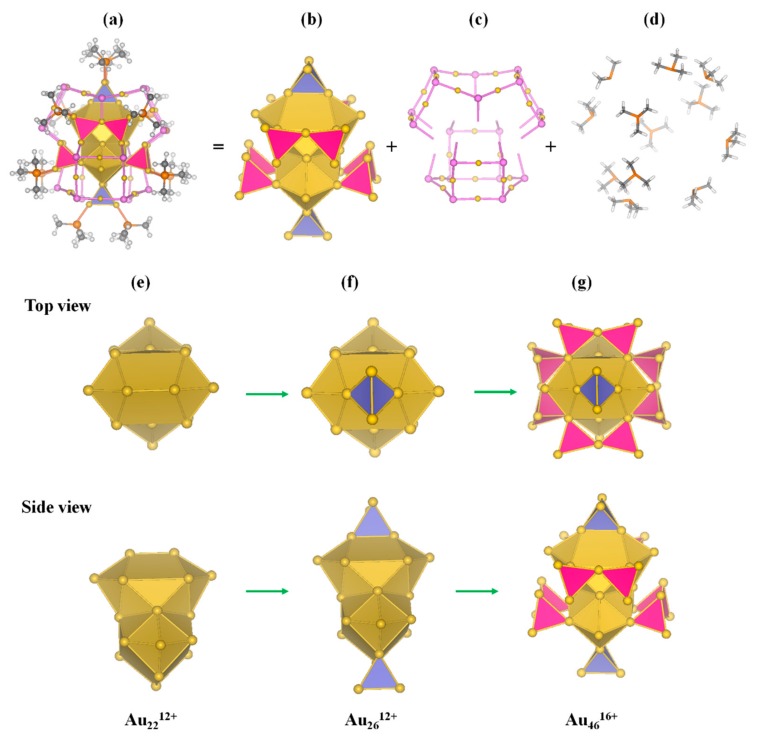
(**a**) Structural model of Au_70_S_20_(PCH_3_)_12_. The [Au_12_(µ_3_-S)_10_]^8−^ and PCH_3_ protecting motifs are given as ball-and-stick models (Au, yellow; P, orange; S, pink; C, gray; H, white). The Au cores are shown as polyhedra. (**b**) Model of Au_46_^16+^ core, (**c**) Two [Au_12_(µ_3_-S)_10_]^8−^ staple motifs, (**d**) Model of twelve PCH_3_ protecting motif, (**e**) Au_22_^12+^ superatomic molecule, (**f**) Au_26_^12+^ core, (**g**) Au_46_^16+^ core.

Based on the divide-and-protect concept [22], different building blocks were tried to find the proper segmentation mode. The cluster can be viewed as Au-core and protecting motifs. Through analysis on the structure, the protecting motifs include twelve separate PCH_3_ and (Au-S)_n_ motifs. According to the segmentation analysis in Supplementary Materials, Au_70_S_20_(PCH_3_)_12_ cluster is divided into three parts as Figure 2 and Figure 3 show. Au_70_S_20_(PCH_3_)_12_ cluster can be written as Au_70_S_20_(PCH_3_)_12_ = [Au_46_^16+^][Au_12_(µ_3_-S)_10_^8−^]_2_[PCH_3_]_12_. The core of the cluster is Au_46_^16+^ with two new [Au_12_(µ_3_-S)_10_]^8−^ staple motifs and 12 PCH_3_ protecting it. The [Au_12_(µ_3_-S)_10_]^8−^ staple motif containing ten µ_3_-S atoms is observed for the first time in Au-L clusters. As shown in Figure 2c and Figure 3c, [Au_12_(µ_3_-S)_10_]^8−^ motif can be easily identified from the cluster. Worth noting is that [Au_12_(µ_3_-S)_10_]^8−^ motif has six branches, which is obviously different from common staple motif and it is unprecedented in Au_m_(SR)_n_ clusters. Each S atom is triply coordinated to the neighboring Au atoms in a µ_3_ bridging form. [Au_12_(µ_3_-S)_10_]^8−^ motif has six S legs, thus we term it six-tooth staple motif. According to the theoretical studies by Jiang et al., other motifs than common staple motifs may exist [42]. Moreover, Au_x_S_y_ unit is theoretically predicted existing in core-shell structures of Au_m_S_n_ clusters [43,44]. [Au_12_S_8_]^4−^ anion presented in the synthesized crystal thioaurate [Ph_4_As]_4_[Au_12_S_8_]. The framework of [Au_12_S_8_]^4−^ anion is a distorted cube, moreover, sulfur, and gold atoms locate at the corners and edge midpoints of the cubic structure, respectively [45].

The Au_3_(µ_3_-S) unit has been proposed as an elementary block and used to design a group of quasi-fullerence hollow-cage [Au_3n_(µ_3_-S)_2n_]^n−^ clusters with high stability [46]. [Au_12_(µ_3_-S)_10_]^8−^ motif can be viewed as a part of [Au_15_(µ_3_-S)_10_]^5−^ cluster, which is a half cage. Here, [Au_12_(µ_3_-S)_10_]^8−^ six-tooth staple motif as a whole protects Au_46_^16+^ core. The configuration of the vertex-sharing Au_7_ core in Au_46_^16+^ core resembles those in Au_28_(SR)_20_ and Au_20_(SR)_16_ clusters [34,47]. From Figure 2, Au_46_^16+^ core is composed of five edge-sharing Au_6_, four vertex-sharing Au_7_ and two Au_4_ superatoms. The five Au_6_ superatoms compose an Au_22_^12+^ kernel. The valence electrons of Au_22_^12+^ core is 10e, which is also a 10e superatomic molecule (Figure 1c and Figure 3).

From Figure 2, we can see that the 12 terminal S legs in two [Au_12_(µ_3_-S)_10_]^8−^ staple motifs connect to the neighboring Au_7_ cores. The two [Au_12_(µ_3_-S)_10_]^8−^ motifs protect Au_46_^16+^ core from both top and bottom sides stabilizing the cluster. The average bond length of Au-S in [Au_12_(µ_3_-S)_10_]^8−^ is 2.39 Å suggesting a covalent single bond. The average bond angle of ∠Au-S-Au is 94.7° and thus deviate only slightly from the ideal 90° expected for bonding involving the sulfur 3p orbitals. Gold attempts to maintain linearity with average bond angle of ∠S-Au-S being 171.4°.

Figure S1 gives Au–Au contacts in the optimized structure of Au_70_S_20_(PCH_3_)_12_ cluster: (a) Au_22_ innermost core is 5 × 2e SAN, (b) Au_22_ innermost core is a 10e-superatomic molecule. Also given are the aurophilic contacts between motifs and superatoms and the aurophilic contacts between superatoms. Noticeable gold–gold interactions (baby blue and black lines in Figure S1a,b, Supporting Materials) between the Au atoms in [Au_12_(µ_3_-S)_10_]^8−^ and neighboring gold cores are present. The Au–Au aurophilic distances range from 2.82–3.01 Å, with the average Au–Au distance being 2.91 Å smaller than the Au–Au van der Waals radii (3.32 Å) [48,49]. The blue lines in Figure S1a label the aurophilic interactions between Au_6_ and Au_4_ cores, and the interactions between Au_4_ cores. The green lines in Figure S1b label the aurophilic interactions between Au_22_ and neighboring Au_4_ cores. The Au–Au distances range from 2.86–3.01 Å, and the average Au–Au distance is 2.93 Å. The short bond distance between Au and Au indicates strong aurophilic interactions. Thus, the interaction mode between six-tooth staple motifs and Au cores includes clamping and aurophilic interactions, which stabilize the Au_70_S_20_(PCH_3_)_12_ cluster. Here, the staple motif can extend to six-tooth mode. The staple motif only includes Au and S elements, which is obviously different from previous staple motifs. From above analysis, we can see that both the position of the six-tooth staple motifs and Au–Au contacts in the cluster dedicate to the stability of Au_70_S_20_(PCH_3_)_12_ cluster.

### 3.2. Chemical Bonding Analysis

**Figure 4:**
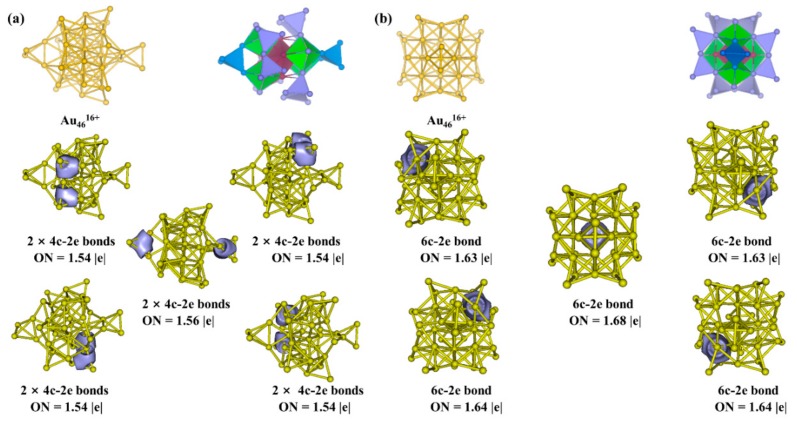
Structures, superatom-network models and adaptive natural density partitioning (AdNDP) localized natural bonding orbitals of (**a**) 4**c**–2**e** bonds (side view), (**b**) 6**c**–2**e** bonds (top view) in Au_46_^16+^ core of Au_70_S_20_(PCH_3_)_12_ cluster.

In order to verify the electronic structure of Au_70_S_20_(PCH_3_)_12_ cluster, we carried out chemical bonding analysis. The electronic structure of the cluster followed the SAN model, that is, it had a network of fifteen 2e-superatoms, abbreviated as 15 × 2e SAN, which contained five 2e-Au_6_ and ten 2e-Au_4_ superatoms. We took the Au_46_^16+^ core out of the cluster separately while keeping the structure identical to that in Au_70_S_20_(PCH_3_)_12_ cluster to analyze the chemical bonds. As expected, AdNDP analysis in Figure 4 indicated that there are 10 four-center-two-electron (4c–2e) bonds with occupancy numbers (ON) = 1.54−1.56 |e|, five 6c−2e bonds with ONs = 1.63−1.68 |e|. Vertex-sharing Au_4_ superatoms were present in the experimentally determined Au_20_(SR)_16_ and Au_36_(SR)_24_ clusters [33,34].

For purposes of confirming the segmentation scheme, the difference of Au–Au distances inside the Au_46_ core and those between Au_46_ core and two six-tooth staple motifs were recorded. Figure S2 (Supporting Materials) displays all the Au–Au distances, which include the distances between Au_46_ core and two six-tooth staple motifs (black dots), the Au–Au distances in Au_22_ core (red dots), in two Au_4_ superatoms on top and bottom of the cluster (blue dots), in the four pairs of vertex-sharing Au_4_ superatoms (purple dots). The average Au–Au distances of the above four groups were 2.90, 2.91, 2.82, and 2.86 Å, respectively. From the figure, we can see that, the Au–Au distances between Au_46_ core and two six-tooth staple motifs and distances in the Au_22_ core were relatively bigger than other two groups. The Au_22_ core was consistent with the former report [21]. The reason for the Au–Au distances in Au_22_ core being relatively bigger are probably that the repulsive interactions of Au atoms can be reduced in this way. Lower repulsion is helpful to form a Au_22_ core. The Au–Au distances in the ten Au_4_ superatoms were shorter than those between the Au-core and staple motifs, which follow the concept of SAN model. The shorter Au–Au distances were helpful to the formation of Au_4_ superatoms. In short, the existence of ten Au_4_ superatoms were reasonable, which has been supported from the viewpoint of Au–Au distances.

**Figure 5:**
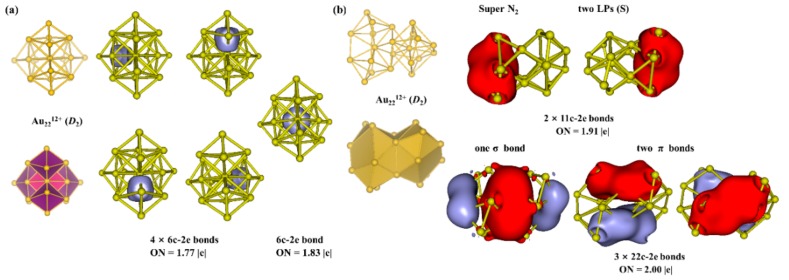
(**a**) Structure, superatom-network model and AdNDP localized natural bonding orbitals of Au_22_^12+^ core. (**b**) Structure, superatomic molecular model and AdNDP localized natural bonding orbitals of Au_22_^12+^ superatomic molecule.

Further analysis of the innermost Au_22_^12+^ core was performed and the structure of Au_22_^12+^ core stayed the same as that in Au_46_^16+^ core. The results are given in Figure 5. From Figure 5a, we can see that Au_22_^12+^ core can be viewed as five edge-sharing Au_6_ superatoms. AdNDP analysis confirms that there are five 6c–2e bonds. ON is 1.83 |e| for the middle 6c–2e bond, while ONs are all 1.77 |e| for the marginal 6c–2e bonds. The Au_9_ kernel in Au_18_(SR)_14_ cluster consists of two Au_6_ superatoms [50,51]. Au_22_^12+^ core has 10 valence electrons which is identical to a N_2_ molecule, and it can be viewed as a super-N_2_ molecule. From Figure 5b, AdNDP analysis demonstrates that Au_22_^12+^ has two 11c–2e super 1S lone pairs with ONs being 1.91 |e|, one 22c–2e super-σ bond and two 22c–2e super π bonds with ONs being 2.00 |e|.

### 3.3. Aromatic Analysis

**Figure 6:**
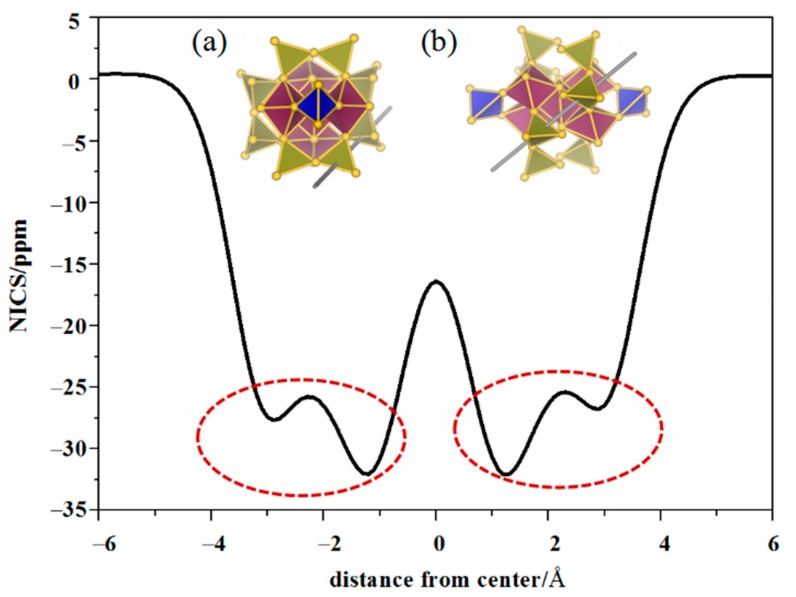
NICScc-scan curve of the Au_46_^16+^ core, which is the scan along the centers of the neighboring Au_4_ superatoms in the range of –6.0–6.0 Å. The red dotted ovals in the figure signal the presence of Au_4_ superatoms. The structures labeled in (**a**) and (**b**) indicate two views of the scan.

NICS-scan method is proposed by Stanger, which is similar to the screen method of aromatic center and has been used to predict the aromatic properties of molecules and clusters [52,53,54]. Here, we use NICS-scan method to further verify the existence of Au_4_ superatoms and we have demonstrated the existence of Au_4_ superatoms in Au_20_(SR)_16_, Au_28_(SR)_20_ and Au_30_S_2_(SR)_18_ clusters in our former work [19,36]. Figure 6 is the NICS-scan curve of Au_46_^16+^ core along the centers of two neighboring Au_4_ superatoms in the range of −6.0–6.0 Å. The position of NICS(0) is set at the midpoint of the geometric centers of two Au_4_ superatoms. Two views of the scan in Au_46_^16+^ have been given in the figure. Considering the symmetry of Au_46_^16+^, we only give one scan curve of the cluster. It is obvious that there are two dotted ovals in the figure, indicating two non-conjugate Au_4_ superatoms, which further support the SAN model. The NICS(0) values of the two Au_4_ superatoms are both −32.2 ppm much smaller than benzene molecule (−9.7 ppm), indicating strong aromaticity. The NICS-scan method is applied to verify Au_4_ superatoms in Au_46_^16+^ core, thus the Au_4_ superatoms are further verified from the aromatic view.

### 3.4. Cu_6_@Au_12_(μ_3_-S)_8_, Ag_6_@Au_12_(μ_3_-S)_8_, and Au_6_@Au_12_(μ_3_-S)_8_ Clusters

Worth noting is that [Au_12_(μ_3_-S)_8_]^4−^ was experimentally crystallized earlier [45]. Meanwhile, the structure of [Au_12_(μ_3_-S)_8_]^4−^ was theoretically studied [46]. We obtained the optimized structure and harmonic frequencies of [Au_12_(µ_3_-S)_8_]^4−^ cluster at the level of PBE0/Lanl2dz(Au), 6-31G *(S). The optimized structure presented a cubic structure in O_h_ symmetry and the Au-S bond length is 2.38 Å. It was found that the harmonic vibrational frequencies of [Au_12_(μ_3_-S)_8_]^4−^ were all positive. The HOMO-LUMO gap was 2.90 eV, further indicating its high stability.

**Figure 7:**
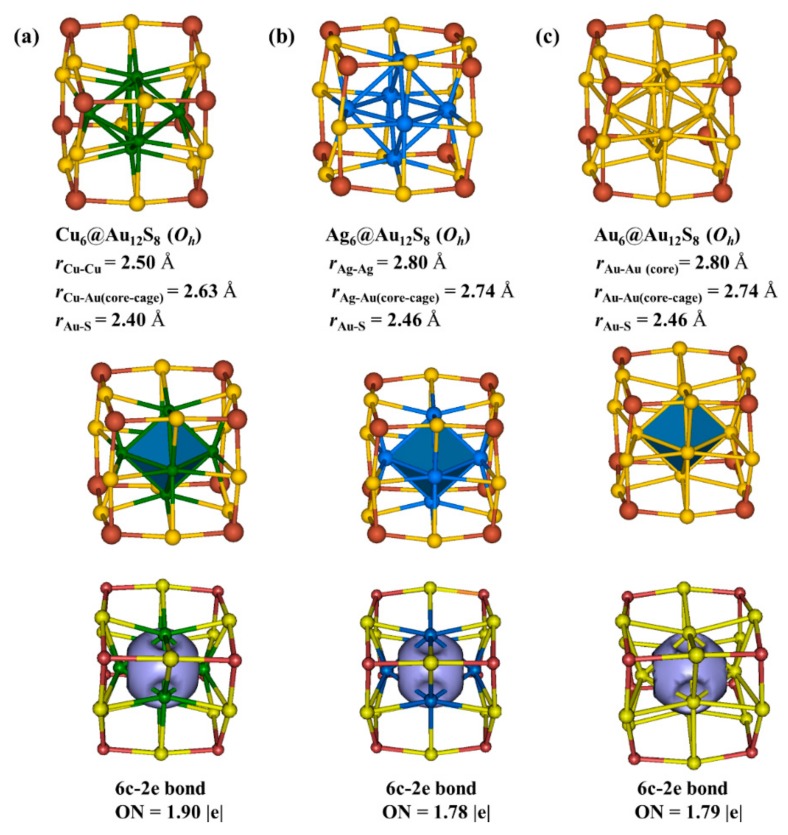
Structures, superatom models and AdNDP localized natural bonding orbitals of 6c-2e bonds in (**a**) Cu_6_@Au_12_(μ_3_-S)_8_, (**b**) Ag_6_@Au_12_(μ_3_-S)_8_, and (**c**) Au_6_@Au_12_(μ_3_-S)_8_ clusters. Cu, green; Ag, blue; Au, yellow; S, brown.

Jiang et al. have predicted several core-in-cage gold sulfide Au_x_S_y_^−^ clusters observed in MALDI fragmentation of Au_25_(SR)_18_^−^ cluster theoretically [42]. They stated that the Au core in the core-in-cage cluster may catalyze reactions. Inspired by the half-cage [Au_12_(μ_3_-S)_10_]^8−^ staple motif, the cubic [Au_12_(μ_3_-S)_8_]^4−^ cluster can be regarded as a cage staple motif. Thus, we designed three core-in-cage clusters, Cu_6_@Au_12_(μ_3_-S)_8_, Ag_6_@Au_12_(μ_3_-S)_8_, and Au_6_@Au_12_(μ_3_-S)_8_. The structures, models and AdNDP analysis of the three designed clusters are collected in Figure 7. The core-in-cage clusters can keep *O_h_* symmetry after relaxation. The harmonic vibrational frequencies of the three clusters are all positive, indicating they are real local minima on potential energy surfaces. The infrared spectrograms (IR) of them are given in Figure S3. The HOMO-LUMO gaps of Cu_6_@Au_12_(μ_3_-S)_8_, Ag_6_@Au_12_(μ_3_-S)_8_, and Au_6_@Au_12_(μ_3_-S)_8_ clusters are 3.59, 2.97, and 2.87 eV, suggesting their high stability. Cu_6_@Au_12_(μ_3_-S)_8_ is more stable than other two clusters because Ag_6_ and Au_6_ are too large. The Cu–Au, Ag–Au and Au–Au distances between the atoms in core and cage of the three clusters are 2.63, 2.74 and 2.74 Å (Figure 7), respectively. All of them are smaller than the sum of their van der Waals radii (3.12, 3.38, and 3.32 Å) [55], demonstrating that Cu–Au, Ag–Au, and Au–Au interactions play a dominant role in stabilizing the clusters. The cores of the designed clusters are all-metal, which are reminiscent of all-metal aromatic. Thus it is necessary to calculate the NICS(0) values to evaluate the stabilities. The NICS(0) values of Cu_6_@Au_12_(μ_3_-S)_8_, Ag_6_@Au_12_(μ_3_-S)_8_, and Au_6_@Au_12_(μ_3_-S)_8_ are −19.6, −17.0, and −17.9 ppm, respectively. The largely negative NICS(0) values of the cores exhibit that they are aromatic and stable. The aromaticity of the centers contributes to the stabilities of the clusters.

In order to study the thermodynamic stability of the Cu_6_@Au_12_(μ_3_-S)_8_, Ag_6_@Au_12_(μ_3_-S)_8_ and Au_6_@Au_12_(μ_3_-S)_8_ clusters, Cu_6_@Au_12_(μ_3_-S)_8_ cluster is taken as a test case. The thermodynamic stabilities of Cu_6_@Au_12_(μ_3_-S)_8_ cluster is further confirmed by ab initio molecular dynamics (AIMD) simulations. The AIMD studies of the cluster is carried out using Vienna ab initio simulation package (VASP) with PBE0 method [39,56]. Four different temperatures at 300, 500, 700, and 1000 K with a simulation time of 8ps have been performed. The AIMD simulations of Cu_6_@Au_12_(μ_3_-S)_8_ cluster are plotted in Figure S4. From the figure, it is obvious that the structure of Cu_6_@Au_12_(μ_3_-S)_8_ cluster can keep after simulation in the temperature range of 300–1000 K, indicating its high thermostability.

The chemical bonding patterns of the three clusters have been analyzed. According to the results of AdNDP analysis (see Figure S5), each M_6_@Au_12_(μ_3_-S)_8_ (M = Cu, Ag, and Au) cluster has 24 2c-2e Au-S σ bonds with ONs being 1.85, 1.83 and 1.82 |e|, respectively. From Figure 7, each cluster has one 6c–2e bond, and occupancy numbers of the three 6c–2e bonds in Cu_6_@Au_12_(μ_3_-S)_8_, Ag_6_@Au_12_(μ_3_-S)_8_ and Au_6_@Au_12_(μ_3_-S)_8_ are 1.90, 1.78, and 1.79 |e|, respectively.

## 4. Conclusions

In conclusion, we have explored the electronic and geometric structure of the recently determined Au_70_S_20_(PPh_3_)_12_ cluster on the basis of the divide-and-protect concept and SAN model. Au_70_S_20_(PPh_3_)_12_ cluster is a 30e-compound, which does not satisfy the magic number of SAC concept. Based on SAN model, the cluster has fifteen 2e-superatoms. The Au_46_^16+^ core is composed of one Au_22_^12+^ innermost core and ten surrounding 2e-Au_4_ superatoms. The Au_22_^12+^ innermost core can either be viewed as a network of five 2e-Au_6_ superatoms, or be considered as a 10e-superatomic molecule. When Au_22_^12+^ innermost core is viewed as a network of five 2e-Au_6_ superatoms, the Au_46_^16+^core can be described as a 15 × 2e SAN consisting of 10 × 2e Au_4_ and 5 × 2e Au_6_ superatoms. The vertex-sharing Au_7_ core exists in the experimentally determined Au_20_(SR)_16_ and Au_36_(SR)_24_ clusters. A new branching staple motif, six-tooth staple motif, [Au_12_(µ_3_-S)_10_]^8−^, is discovered in Au-L clusters for the first time. The six-tooth staple motif is obviously different from common staple motifs, which have six S legs. Here the newly discovered staple motif enriches the staple motif family. The NICS-san method has been used to confirm the presence of Au_4_ superatoms. The new segmentation method here can properly explain the structure and stability of Au_70_S_20_(PPh_3_)_12_ cluster. The reason for the stability and the nature of bonds have been given. Concretely, the six-tooth staple motifs, the superatom network, the aromatic of the superatoms and Au–Au interactions contribute to the stability of the cluster. We have designed three core-in-cage Cu_6_@Au_12_(μ_3_-S)_8_, Ag_6_@Au_12_(μ_3_-S)_8_, and Au_6_@Au_12_(μ_3_-S)_8_ clusters based on [Au_12_(μ_3_-S)_8_]^4−^. The three clusters are stable in O_h_ symmetry. Each of them has one 6c-2e bond in the core. Aromatic analysis reveals that they are aromatic molecules. The [Au_12_(μ_3_-S)_8_]^4−^ cluster has been experimentally synthesized, and the three constructed clusters are stable based on our computation, thus the three designed clusters may be synthesized in future. Our work will provide some new perspectives to the electronic structure and stability of Au_70_S_20_(PPh_3_)_12_ cluster. The concept of half-cage and cage staple motif could offer some reference to future synthesis of Au-L clusters.

## Supplementary Materials

The following are available online at https://www.mdpi.com/2079-4991/9/8/1132/s1, The segmentation method of Au_70_S_20_(PCH_3_)_12_ cluster. Figure S1: The Au–Au contacts in the optimized structure of Au_70_S_20_(PCH_3_)_12_ cluster. (a) Au_22_ innermost core is 5 × 2e SAN, (b) Au_22_ innermost core is a 10e-superatomic molecule. The baby blue and black lines in the structure indicate aurophilic contacts between motifs and superatoms, while blue and green lines show aurophilic contacts between superatoms. Figure S2: (a) The Au–Au bond distances between Au_46_ core and staple motifs, (b) the Au–Au distances in Au_22_ core, (c) the Au–Au distances in two Au_4_ superatoms on top and bottom of the cluster, (d) the Au–Au distances in the four pairs of vertex-sharing Au_4_ superatoms. Figure S3: IR spectra of Cu_6_@Au_12_(μ_3_-S)_8_, Ag_6_@Au_12_(μ_3_-S)_8_ and Au_6_@Au_12_(μ_3_-S)_8_ clusters. Figure S4: Geometric configuration of Cu_6_@Au_12_(μ_3_-S)_8_ at after 8 ps AIMD simulations at (a) 300 K, (b) 500 K, (c) 700 K and (d) 1000 K, respectively. Cu, green; Au, yellow; S, brown. Figure S5: Geometries (Cu, green; Ag, blue; Au, yellow) and AdNDP localized natural bonding orbitals of Au-S σ-bonds in (a) Cu_6_@Au_12_(μ_3_-S)_8_, (b) Ag_6_@Au_12_(μ_3_-S)_8_ and (c) Au_6_@Au_12_(μ_3_-S)_8_ clusters.
