# Supplementary material for: New Perspectives on the Electronic and Geometric Structure of Au70S20(PPh3)12 Cluster: Superatomic-Network Core Protected by Novel Au12(µ3-S)10 Staple Motifs
Source: Nanomaterials (Basel). 2019 Aug 6;9(8):1132. doi: 10.3390/nano9081132 (PMC6722785; doi:10.3390/nano9081132)
Supplement: Supplementary file 1 [file nanomaterials-09-01132-s001.pdf]

Supplementary Materials

New Perspectives on the Electronic and Geometric Structure of $Au_{70}S_{20}(PPh_3)_{12}$ Cluster: Superatomic-network Core Protected by Novel $Au_{12}(\mu_3\text{-}S)_{10}$ Staple Motifs

Zhimei Tian,[1,2] Yangyang Xu,[3] Longjiu Cheng*[1,4]

1 Department of Chemistry, Anhui University, Hefei 230601, Anhui, China; clj@ustc.edu (L.C)

2 School of Chemistry and Materials Engineering, Fuyang Normal University, Fuyang 236037, Anhui, China; thzm@fync.edu.cn (Z.T.)

3 School of Social and Public Administration, East China University of Science and Technology, Shanghai 200237, China; gogoundok_cool@126.com (Y.X.)

4 Anhui Province Key Laboratory of Chemistry for Inorganic/Organic Hybrid Functionalized Materials, Anhui University, Hefei 230601, Anhui, China

* Correspondence: clj@ustc.edu (L.C.); Tel.: +86-0551-63861279 (L.C.)

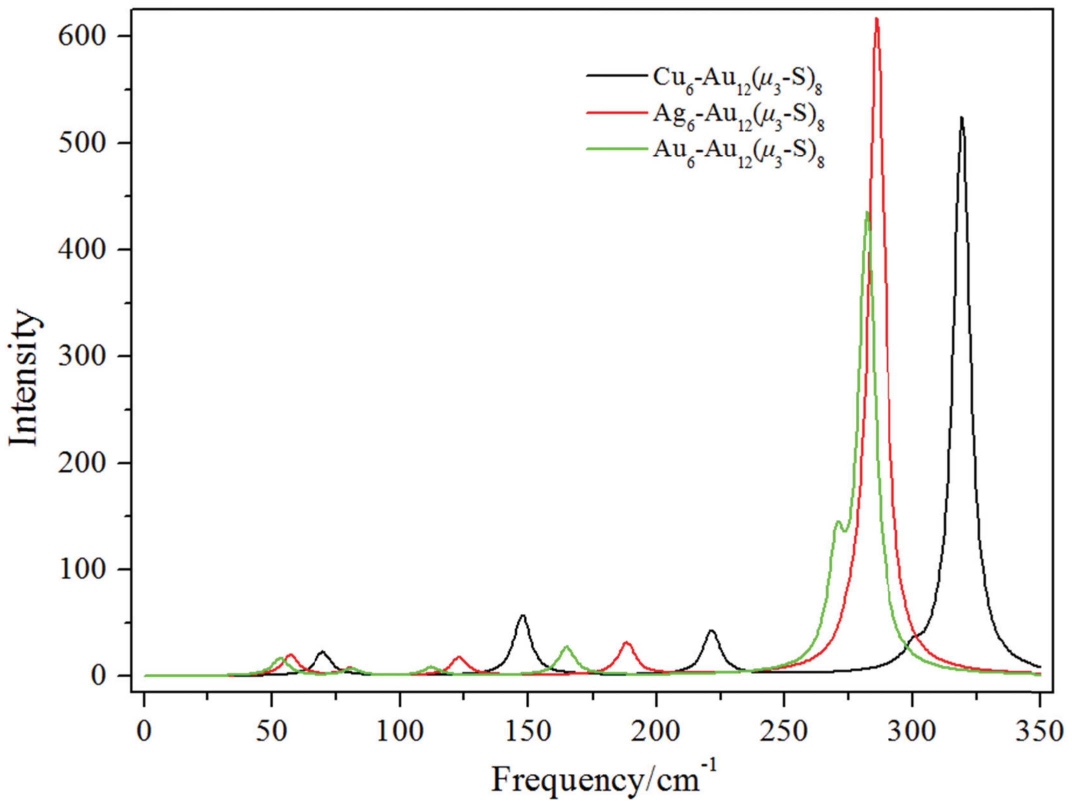

Figure S1. IR spectra of Cu_{12}-$Au_{12}(\mu_3$-$S)_8$, Ag_6-$Au_{12}(\mu_3$-$S)_8$ and Au_6-$Au_{12}(\mu_3$-$S)_8$ clusters.

(a)

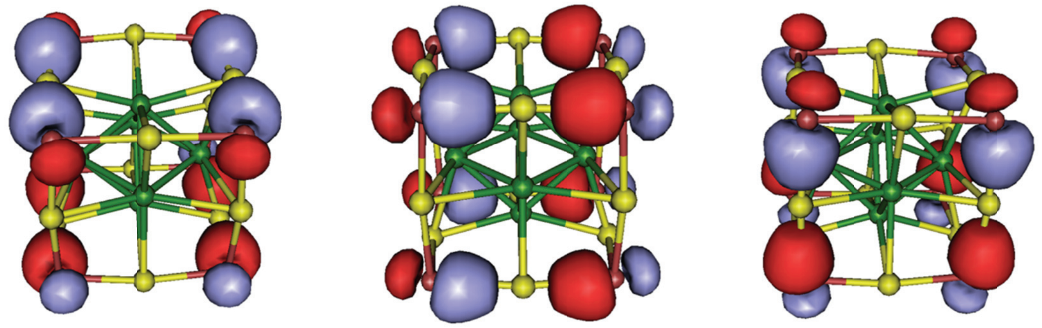

Cu_6-$Au_{12}S_6$ (O_h)
24 × 2c-2e Au-S σ-bonds
ON = 1.85 |e|

(b)

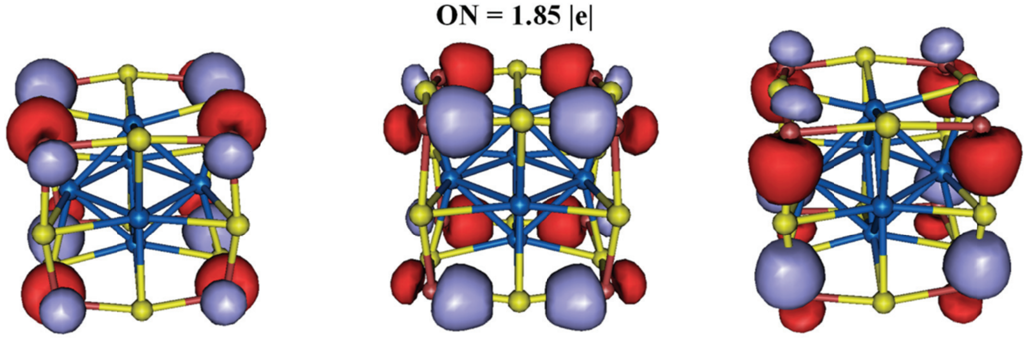

Ag_6-$Au_{12}S_6$ (O_h)
24 × 2c-2e Au-S σ-bonds
ON = 1.83 |e|

(c)

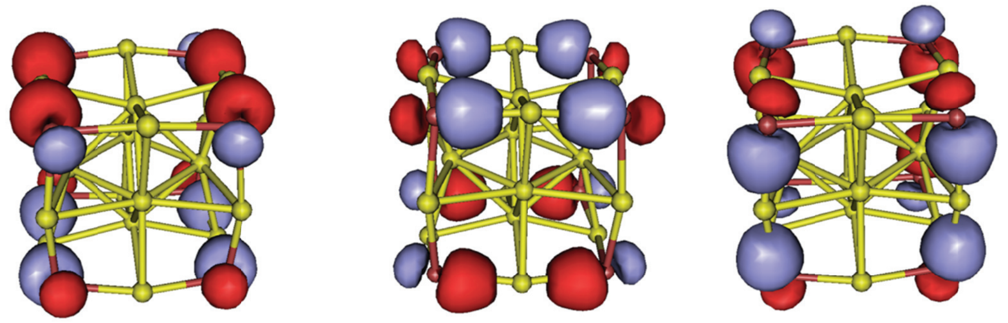

Au_6-$Au_{12}S_6$ (O_h)
24 × 2c-2e Au-S σ-bonds
ON = 1.82 |e|

Figure S2. Geometries (Cu, green; Ag, blue; Au, yellow) and AdNDP localized natural bonding orbitals of Au-S σ-bonds in (a) Cu_{12}-(μ_3-S)$_8$, (b) Ag_6-Au_{12}(μ_3-S)$_8$ and (c) Au_6-Au_{12}(μ_3-S)$_8$ clusters.
